# Impact of thyroid cancer on the cancer risk in patients with non-alcoholic fatty liver disease or dyslipidemia

**DOI:** 10.1038/s41598-023-28112-2

**Published:** 2023-01-19

**Authors:** Joon Ho, Eunhwa Kim, Myeongjee Lee, Inkyung Jung, Young Suk Jo, Jandee Lee

**Affiliations:** 1 Open NBI Convergence Technology Research Laboratory, Department of Surgery, Yonsei University College of Medicine, Seoul, South Korea; 2 Biostatistics Collaboration Unit, Department of Biomedical Systems Informatics, Yonsei University College of Medicine, Seoul, South Korea; 3 Division of Biostatistics, Department of Biomedical Systems Informatics, Yonsei University College of Medicine, Seoul, South Korea; 4 Open NBI Convergence Technology Research Laboratory, Department of Internal Medicine, Yonsei University College of Medicine, Seoul, South Korea

**Keywords:** Thyroid cancer, Risk factors

## Abstract

The raised prevalence of obesity has increased the incidence of obesity-related metabolic diseases such as dyslipidemia (DL) and non-alcoholic fatty liver disease (NAFLD), along with the development and progression of various types of cancer, including thyroid cancer. In this study, we investigated whether thyroid cancer in patients with DL and NAFLD could be a risk factor for other cancers. To achieve our goal, we generated two independent cohorts from our institution and from the National Health Insurance System in South Korea. Based on the ICD-10 code, we conducted exact matching (1:5 matching) and estimated the overall risk of thyroid cancer for other cancers in patients with DL or NAFLD. Univariate and multivariate analyses showed that the hazard ratio (HR) of thyroid cancer was 2.007 (95% Confidence Interval [CI], 1.597–2.522) and 2.092 (95% CI, 1.546–2.829), respectively in the institutional cohort and 1.329 (95% CI, 1.153–1.533) and 1.301 (95% CI, 1.115–1.517), respectively in the nationwide cohort. Risk analysis revealed a significant increase in the HR in lip, tongue, mouth, lung, bone, joint, soft tissue, skin, brain, male cancers and lymphoma after thyroid cancer occurred. Thyroid cancer in patients with DL or NAFLD might be a valuable factor for predicting the development of other cancers.

## Introduction

The prevalence of obesity has increased worldwide over the past few decades. This increase in obesity causes various obesity-related metabolic diseases such as dyslipidemia (DL), non-alcoholic fatty liver disease (NAFLD), and diabetes. Furthermore, obesity is associated with the risk of certain types of malignancies, including breast cancer, colon cancer, and liver cancer. Because of the high prevalence of obesity-related metabolic diseases and cancer, it is frequently observed that these diseases and cancers occur in succession at different times in the same patients^1–4^. It is known that obesity-related metabolic diseases contribute to the development and progression of cancers. For example, NAFLD can cause malignant tumors such as hepatocellular carcinoma^5–7^. Several studies have reported that DL increases the risk of cancers, such as non-small cell lung cancer and colorectal cancer^8,9^. Rice et al*.*^10^ reported that high cholesterol status is associated with the risk of developing prostate cancer.

Thyroid cancer is the most common endocrine cancer, and the number of patients with thyroid cancer worldwide has rapidly increased over the past 20 years^11–15^. Although this increase has been caused by the introduction of high-sensitivity ultrasound to population-based screening, an association between thyroid cancer and obesity has also been suggested in terms of the etiological aspect^16–20^. Furthermore, patients with DL and thyroid cancer simultaneously, showed a higher risk of second primary cancer than patients with thyroid cancer and no DL did. As patients with thyroid cancer usually have a good prognosis, characterized by a long survival period, and the age at diagnosis is relatively younger than that of patients with other types of malignancies, the issue related to second primary cancer is particularly important. Recent population-based studies have suggested that the risk of second primary cancer is higher in survivors of thyroid cancer than in the general population^21–23^.

From this perspective, this study examined whether thyroid cancer can be used as a predictor of the development of other primary cancers in patients with metabolic diseases^24^. To achieve this goal, we investigated the effect of antecedent thyroid cancer on the risk of other primary cancers in patients with DL and NAFLD. To validate our results, two cohorts from our institution and a nationwide database were independently analyzed using nested case–control studies with propensity score matching.

## Results

### Risk estimation of cancer in patients with NAFLD or DL according to the presence of thyroid cancer using our institutional cohort.

**Figure 1:**
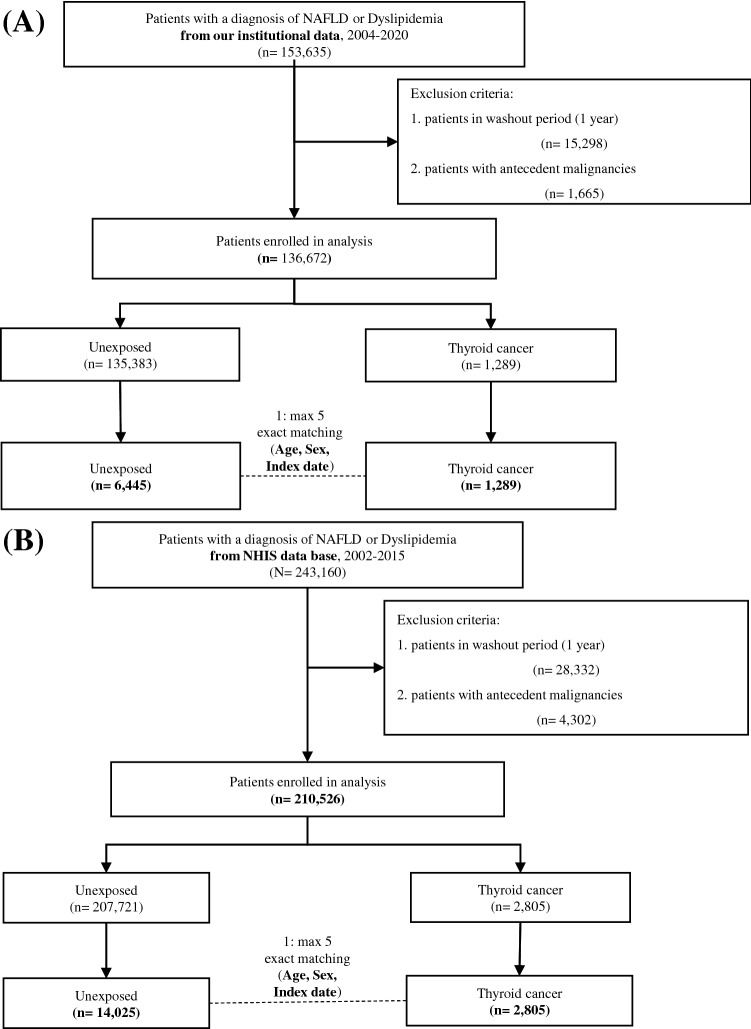
Schematic flow of study design using our institutional cohort (**A**) and Korean national cohort (**B**). Abbreviations: NAFLD, non-alcoholic fatty liver disease; NHIS, national health insurance system.

Based on our inclusion and exclusion criteria, 136,672 patients were finally included (Fig. 1A). Next, we classified our institutional cohort into three groups: patients with NAFLD or DL, patients with NAFLD only, and patients with DL only. The baseline characteristics of our institutional cohort are summarized in Supplementary Table 1. Briefly, 15,907 patients with NAFLD and 112,088 patients with DL were selected, and 1289 patients with NAFLD or DL developed thyroid cancer. The mean age of patients with NAFLD or DL was 52.8 ± 12.1 years, and the average follow-up period was 85.7 ± 59.5 months.

The propensity scores for age, sex, and index date were matched one-to-maximum five with patients without thyroid cancer in the total patient group, and 6445 patient groups were extracted (Fig. 1A and Supplementary Table 2). Although anthropometric variables such as body mass index (BMI) and waist circumference did not differ between the groups, patients with thyroid cancer had higher total cholesterol and lower AST, ALT, and gamma-glutamyl transpeptidase (γGT) levels. Next, the effects of each variable measured in the patient group on the occurrence of other primary cancers were analyzed. Univariate analysis showed that glucose and thyroid cancer increased the hazard ratio (HR) for cancer risk in the three cohorts (Supplementary Table 3). For patients with NAFLD only, γGT also increased the HR, but the results were not consistent in the other two cohorts. Total and LDL cholesterol decreased the HR in patients with NAFLD or DL, but this result was not consistent in the other two groups. Multivariate analysis showed that only thyroid cancer had a statistically significant effect on the HR in all three cohorts (Supplementary Table 4). As our data indicated that the presence of thyroid cancer in patients with NAFLD or DL increased the risk of other primary cancers, we compared the risks of individual cancers according to the presence or absence of thyroid cancer in this cohort (Supplementary Table 5). The overall risk of cancer was higher in patients with thyroid cancer (127 out of 1289 cases, 9.85%) than in those without thyroid cancer (318 out of 6445, 4.93%), indicating that the HR was 2.007 (95% confidence interval [CI], 1.597–2.522). In the risk estimation of individual cancer types, HRs increased in lung, bronchus, bone, joints, soft tissue cancers, and leukemia.

### Risk estimation of cancer in patients with NAFLD or DL according to the presence of thyroid cancer using the Korean national cohort

In fact, our institutional cohort showed a small number of cancer occurrences among patients with thyroid cancer. We used a population-based big data (NHIS) to validate our findings and investigate the risk estimation of individual cancers. Between 2002 and 2015, 243,160 patients with NAFLD or DL were identified. Among them, 210,526 patients were excluded based on our exclusion criteria. As with the analysis of our institutional cohort, 1:5 exact matching was conducted on 2805 patients with thyroid cancer and 14,025 patients without thyroid cancer, whose age, sex, and index year were matched (total 16,830 patients, Fig. 1B). The baseline characteristics of the patients in this cohort and the presence of thyroid cancer were related to a higher BMI (Table 1). However, other chemical variables, such as glucose, total cholesterol, triglyceride, HDL cholesterol, LDL cholesterol, were also measured. The AST and γGT were higher in patients without thyroid cancer.

**Table 1:** Baseline characteristics in patients with NAFLD or dyslipidemia according to thyroid cancer in Korean national cohort after matching.

	Thyroid cancer		*P*-value
	Absence (n = 14,025)	Presence (n = 2805)	
Sex			
Male	2,630 (18.75)	526 (18.75)	
Female	11,395 (81.25)	2,279 (81.25)	
BMI	24.89 ± 3.13	25.03 ± 3.10	0.0275
Waist circumference	82.57 ± 8.23	82.82 ± 8.32	0.1226
SBP	135.77 ± 18.12	135.17 ± 17.33	0.1257
DBP	85.14 ± 11.20	85.08 ± 10.85	0.8409
Glucose	110.29 ± 37.45	108.29 ± 37.04	0.0069
Total cholesterol	229.09 ± 40.72	227.27 ± 40.48	0.0260
Triglyceride	164.07 ± 101.66	154.42 ± 93.25	< .0001
HDL	51.37 ± 20.52	50.26 ± 11.94	< .0001
LDL	140.24 ± 43.37	135.83 ± 40.60	< .0001
Hb	13.95 ± 1.31	14.00 ± 1.32	0.0616
Cr	1.03 ± 1.21	0.98 ± 0.93	0.0600
AST	31.39 ± 22.71	30.17 ± 16.58	0.0059
ALT	31.75 ± 28.40	31.40 ± 23.14	0.6431
γGT	37.44 ± 47.30	33.60 ± 34.07	0.0001
Urine protein	1.20 ± 0.64	1.23 ± 0.66	0.0928

*BMI* body mass index, *SBP* systolic blood pressure, *DBP* diastolic blood pressure, *HDL* high density lipoprotein, *LDL* low density lipoprotein, *Hb* hemoglobin, *Cr* creatinine, *AST* aspartate transaminase, *ALT* alanine transaminase, *γGT* gamma-glutamyl transpeptidase.

Univariate analysis confirmed that the levels of SBP, Hb, AST, GGT, and the occurrence of thyroid cancer increased the risk of second primary cancer in the entire patient group. Regarding LDL levels, the risk of second primary cancer was reduced in the entire patient group. In the NAFLD patient group, SBP, DBP, Hb, and thyroid cancer levels increased the risk of second primary cancer. In the DL patient group, Hb and thyroid cancer increased the risk of second primary cancer, and total cholesterol and LDL numerical values reduced the risk of second primary cancer (Table 2).

**Table 2:** Cancer risk estimation by univariable stratified Cox regression analysis result after exact matching in Korean national cohort.

	HR (95% CI)		
	NAFLD or DL	NAFLD	Dyslipidemia
BMI	0.992 (0.972–1.012)	0.997 (0.973–1.021)	0.991 (0.970–1.012)
Waist circumference	1.000 (0.993–1.008)	1.004 (0.994–1.014)	1.001 (0.993–1.009)
SBP	1.004 (1.001–1.007)	1.007 (1.003–1.011)	1.003 (0.999–1.006)
DBP	1.003 (0.998–1.009)	1.009 (1.002–1.016)	1.001 (0.995–1.007)
Glucose	1.001 (0.999–1.002)	1.000 (0.998–1.002)	1.001 (0.999–1.002)
Total cholesterol	0.997 (0.996–0.999)	0.998 (0.996–1.000)	0.997 (0.996–0.999)
Triglyceride	0.999 (0.998–1.000)	0.999 (0.998–1.000)	0.999 (0.998–1.000)
HDL	1.002 (0.999–1.005)	1.002 (0.999–1.005)	1.000 (0.997–1.004)
LDL	0.995 (0.992–0.998)	0.996 (0.993–1.000)	0.996 (0.993–0.999)
Cr	1.018 (0.971–1.068)	1.013 (0.951–1.078)	1.006 (0.953–1.062)
Hb	1.082 (1.031–1.135)	1.102 (1.035–1.173)	1.071 (1.015–1.130)
AST	1.002 (1.001–1.004)	1.002 (1.000–1.003)	1.002 (1.000–1.004)
ALT	1.001 (1.000–1.003)	1.000 (0.997–1.003)	1.000 (0.998–1.003)
γGT	1.002 (1.001–1.003)	1.002 (1.000–1.003)	1.001 (1.000–1.002)
Urine protein	1.001 (0.911–1.100)	0.906 (0.776–1.058)	1.016 (0.922–1.120)
Thyroid cancer (ref = unexposed)	1.329 (1.153–1.533)	1.351 (1.112–1.642)	1.375 (1.182–1.600)

*BMI* body mass index, *SBP* systolic blood pressure, *DBP* diastolic blood pressure, *HDL* high density lipoprotein, *LDL* low density lipoprotein, *Hb* hemoglobin, *Cr* creatinine, *AST* aspartate transaminase, *ALT* alanine transaminase, *γGT* gamma-glutamyl transpeptidase.

In the multivariate analysis, only Hb level and thyroid cancer incidence increased the risk of second primary cancer in the NAFLD patient group, DL patient group, and overall patient group (Table 3).

**Table 3:** Cancer risk estimation by multivariable stratified Cox regression analysis result after exact matching in Korean national cohort.

	HR (95% CI)		
	NAFLD or DL	NAFLD	Dyslipidemia
BMI	0.978 (0.947–1.009)	0.975 (0.937–1.015)	0.976 (0.943–1.010)
Waist circumference	1.005 (0.994–1.017)	1.009 (0.994–1.024)	1.007 (0.995–1.020)
SBP	1.007 (1.001–1.013)	1.009 (1.001–1.016)	1.006 (1.000–1.012)
DBP	0.993 (0.983–1.002)	0.997 (0.984–1.010)	0.992 (0.982–1.002)
Glucose	1.000 (0.999–1.002)	1.000 (0.998–1.002)	1.000 (0.999–1.002)
Total cholesterol	0.998 (0.996–1.001)	0.998 (0.995–1.002)	0.999 (0.996–1.001)
Triglyceride	0.999 (0.998–1.000)	0.999 (0.998–1.000)	0.999 (0.998–1.000)
HDL	1.002 (0.998–1.006)	1.003 (1.000–1.006)	1.000 (0.996–1.005)
LDL	0.996 (0.993–1.000)	0.998 (0.993–1.003)	0.997 (0.993–1.001)
Cr	1.009 (0.960–1.061)	1.002 (0.934–1.075)	0.998 (0.944–1.056)
Hb	1.075 (1.015–1.138)	1.086 (1.009–1.169)	1.070 (1.004–1.140)
AST	1.003 (1.000–1.005)	1.004 (1.001–1.007)	1.003 (1.000–1.006)
ALT	0.998 (0.994–1.002)	0.994 (0.989–1.000)	0.997 (0.992–1.001)
γGT	1.001 (1.000–1.002)	1.002 (1.000–1.003)	1.000 (0.999–1.002)
Urine protein	1.010 (0.916–1.115)	0.891 (0.758–1.047)	1.035 (0.935–1.145)
Thyroid cancer (ref = unexposed)	1.301 (1.115–1.517)	1.331 (1.079–1.642)	1.350 (1.147–1.590)

*BMI* body mass index, *SBP* systolic blood pressure, *DBP* diastolic blood pressure, *HDL* high density lipoprotein, *LDL* low density lipoprotein, *Hb* hemoglobin, *Cr* creatinine, *AST* aspartate transaminase, *ALT* alanine transaminase, *γGT* gamma-glutamyl transpeptidase.

Overall, other types of primary cancers occurred in 8.31% of patients with thyroid cancer and 6.37% of patients without thyroid cancer. When thyroid cancer occurred, the risk of cancer was increased compared to that in patients without thyroid cancer in patients with NAFLD or DL (HR, 1.329; 95% CI, 1.153–1.533). In this cohort, thyroid cancer increased the risk of lip, tongue, mouth, lung, bone, joints, soft tissue, skin, brain, male cancer, and lymphoma (Table 4).

**Table 4:** Risk estimation of cancer in patients with NAFLD or dyslipidemia according to thyroid cancer in Korean national cohort.

	Thyroid cancer (N = 16,820)		
	Absence (n = 14,015)	Presence (n = 2805)	
Cancer type	No. of cases (%)	No. of cases (%)	HR (95% CI)
Overall	893 (6.37%)	233 (8.31%)	1.301 (1.115–1.517)
Lip & Tongue & Mouth	3 (0.33%)	1 (0.42%)	3.947 (1.044–14.921)
Stomach	123 (13.77%)	19 (8.15%)	0.737 (0.441–1.233)
Small intestine	1 (0.11%)	1 (0.42%)	5.622 (0.156–202.855)
Colon	81 (9.07%)	12 (5.15%)	0.775 (0.409–1.469)
Liver	92 (10.30%)	11 (4.72%)	0.646 (0.333–1.255)
Pancreas	63 (7.05%)	7 (3.00%)	0.487 (0.210–1.127)
Lung, bronchus	106 (11.87%)	35 (15.02%)	1.641 (1.083–2.487)
Thymus, mediastinum, heart	4 (0.44%)	5 (2.14%)	4.814 (0.818–28.351)
Bone, joints & soft tissue	18 (2.02%)	11 (4.72%)	3.400 (1.605–7.199)
Skin, Melanoma	18 (2.02%)	8 (3.43%)	2.599 (1.084–6.233)
Kidney	28 (3.14%)	8 (3.43%)	1.085 (0.443–2.657)
Brain, CNS	32 (3.58%)	21 (9.01%)	3.154 (1.675–5.940)
Lymphoma	15 (1.68%)	8 (3.43%)	2.716 (1.109–6.649)
Multiple myeloma	10 (1.12%)		
Leukemia	6 (0.66%)	1 (0.42%)	0.692 (0.071–6.772)
Male cancer	55 (6.16%)	22 (9.44%)	1.940 (1.183–3.182)
Female cancer	164 (18.36%)	31 (13.30%)	0.866 (0.571–1.314)
Others	156 (17.47%)	43 (18.45%)	1.394 (0.963–2.017)

HR, hazard ratio; Total (%) = other primary cancer pts. / total pts. Cancer type (%) = specific type pts./other primary cancer pts.

## Discussion

This study showed that the occurrence of thyroid cancer in patients with DL or NAFLD increases the risk of other primary cancers. To validate our results, we used two independent cohorts, an institutional cohort and a nationwide cohort, and to exclude lead-time bias, we conducted case–control studies by propensity score matching.

Previous studies have reported an increased incidence of cancer in patients with DL. Uzunlulu et al. reported that low HDL cholesterol levels were associated with an increased incidence of lung cancer and LDL cholesterol levels were associated with the development of hematologic malignancy^25^. Kim et al.^26^ reported that high triglyceride and low HDL levels were associated with an increase in the incidence of prostate cancer. Kitahara et al.^27^ reported that high total cholesterol levels could increase the risk of breast, colon, and prostate cancers. Many studies have been conducted to explain the relationship between DL and cancer development^28–30^. Studies have reported that a high-lipid diet triggers tumor growth and metastasis^28,31^. Abundant lipid droplets (LD) and high cholesterol ester content in tumor cells have been reported as predictors of cancer aggressiveness^30,32–34^. Another possible mechanism is that cholesterol is an essential component of cell membranes and can affect various signaling pathways, such as cell survival kinases, including AKT serine/threonine kinase/protein kinase B (AKT/PKB)^35,36^. Metabolic remodeling is a hallmark of cancers, and functional tumor-initiating cholesterol-related pathways during a series of remodeling processes have been widely recognized^31,37,38^. With regard to cancer prevention, lipid-lowering agents such as statins (3-hydroxy-3-methyl-glutaryl-coenzyme A reductase and HMG-CoA reductase) have been shown to inhibit the formation and proliferation of metastatic cancer cells^39,40^.

Based on these clinical and experimental studies, obesity-related metabolic diseases, including DL and NAFLD, have been firmly regarded as risk factors for cancer^17,25^. For risk stratification, the severity of DL and noninvasive quantitative anthropometric measurements have been extensively investigated^16^. However, our data indicated that anthropometric measurements, such as BMI and waist circumference, did not increase the HR for second primary cancers in patients with DL or NAFLD, even though the baseline characteristics of nationwide cohort showed that patients with thyroid cancer had a higher BMI value. Moreover, although fasting glucose in our institutional cohort and Hb in the nationwide cohort increased the risk of other primary cancers, the degree of HR increase was not remarkable, and the increase was inconsistent across both cohorts. Laboratory results, including lipid profile and liver function tests at the time of NAFLD or DL diagnosis, were collected. The differences in AST and γGT values between patients with or without thyroid cancer were observed, suggesting the differences in the severity of DL/NAFLD in both groups. However, multivariable analysis did not indicate statistical significance, except γGT, in patients with NAFLD in our institutional cohort. In fact, our study only included patients with DL/NAFLD, but not patients without DL/NAFLD. Therefore, we postulated that all of the study subjects had abnormalities in lipid metabolism, suggesting a relatively lower risk compared to the risk obtained from the patients without DL/NAFLD.

From a molecular biological perspective, cancers develop by driver gene mutations and can be classified by the mechanism of the occurrence of oncogene mutations^41^. First, hereditary factors were demonstrated in twin studies and the genes responsible for cancer predisposition syndromes were identified. Second, epidemiological studies have investigated environmental factors. However, the proportion of oncogene mutations generated by these two classical factors is not sufficiently high to explain the increase in overall cancer incidence. Finally, DNA replication errors (replicative factors), which randomly occur during cell division, are the most common cause of oncogene mutations. Replicative factors can be observed in various types of cancers including the brain, thyroid, breast, liver, kidney, colon, ureter, and bladder. Moreover, leukemia and non-Hodgkin’s lymphoma have driver mutations related to errors in DNA replication^42^. In this etiological aspect of oncogene mutations, we postulated that the ability to prevent replication errors resulting from cell divisions in tissue might be an important defensive mechanism in cancer development, which might differ from person to person. Based on this idea, the development of thyroid cancer in patients with DL or NAFLD, especially in middle-aged patients, might be a signature event that indicates an error-prone situation in later life. DL and NAFLD can increase the risk of second primary cancers in patients with thyroid cancer^43^. In contrast, as shown in this study, the occurrence of thyroid cancer in patients with DL and NAFLD may be related to other primary cancers developed by replicative factors.

In terms of replicative factors, oxidative stress might have an important role in NAFLD and carcinogenesis. In the case of NAFLD, the so-called “two-hit” model in the development and progression has been well-established. The first hit is made by insulin resistance and lipid accumulation, resulting in simple hepatic steatosis. The inflammatory cytokine cascade is activated due to a second hit by oxidative stress or depletion of ATP^44^. In thyroid glands, thyroid hormone synthesis requires H_2_O_2_, suggesting potential exposure to oxidative stress, promoting oxidative DNA damage^45^. Therefore, oxidative stress also might have a role in the development and progression of thyroid cancer^46^. Recent studies have also suggested that the leakage of endotoxins into systemic circulation is also able to promote the inflammation of liver. In particular, endotoxins release pro-inflammatory cytokines from the liver via inflammatory signaling pathways, such as NFκB activation^47^. This pro-inflammatory milieu of systemic circulation might be related to carcinogenesis in diverse organ directly and indirectly with insulin resistance and hyperinsulinemia. Taken together, patients with thyroid cancer might be more exposed to oxidative stress, and the risk of neoplasia-preceding in other organs might also be increased. Moreover, it can be postulated that the risk of second primary cancer in patients with DL/NAFLD might be increased by oxidative stress by various factors, including endotoxins.

To the best of our knowledge, this is the first study to investigate whether thyroid cancer is a risk factor for other primary cancers in patients with DL and NAFLD. Nevertheless, this study had several limitations. First, due to the characteristics of the data, not all test values exist at all times in patients, so the evaluation of the effect of differences in test values on the occurrence of other primary cancers may be inadequate. Since the corresponding population-based big data are built on the basis of diagnosis and medical records, all test results of patients cannot be included. Among the test values of the patient, the values closest to the diagnosis time were used, and factors with few missing values were used for analysis; therefore, there was no difficulty in deriving statistical results. However, there are some limitations to fully reflecting the state of fat metabolism. Second, the follow-up period was relatively short. There was no problem in the comparative analysis by matching the date of diagnosis of metabolic disease between patients with thyroid cancer and those who did not. However, since it was claims data, there may be a limitation in that the follow-up period of a patient with a recent diagnosis date was relatively short. Third, although we excluded patients with various liver diseases to precisely extract NAFLD, it is possible that some patients with diseases such as alcoholic hepatitis were included. However, we thought that not many cases like this were included, as it is difficult to claim insurance in South Korea without including the exact disease name. Fourth, drugs used for dyslipidemia such as statins, which have a potential to affect cancer development and progression, were not investigated. Our data showed that patients with thyroid cancer had lower LDL-cholesterol, suggesting those patients might take statins more frequently. However, as univariate and/or multivariate Cox regression analyses did not show statistical significance, we thought that the effect of statins in our study was not remarkable. Finally, our analysis did not include the difference of therapeutic modalities, such as radioactive iodine therapy (RAIT), which can increase the risk of certain types of malignancy. However, the risk estimation of individual types of cancer did not present different features in representative RAIT-related cancers (e.g., leukemia).

In conclusion, thyroid cancer increases the risk of developing other primary cancers in patients with DL and NAFLD. To improve our understanding of cancer development and prevention, more accumulated population-based data and molecular biological studies are required.

## Methods

### Institutional cohort

For the generation of the cohort from our institution, data from patients aged 20–79 years were extracted using the in-hospital patient information search program. Data were extracted according to the diagnosis, and included patients from September 2004 to 2020, when medical records were used. All in-hospital patients with confirmed diagnoses were included. In the case of DL, the diagnosis was used as is. However, in the case of NAFLD, patient groups were extracted using the inclusion and exclusion ICD 10 code, as described in Supplementary Fig. 1^48^. In cases of thyroid cancer and other types of cancer, patients with a corresponding diagnosis were included. After a washout period of one year, patients were extracted from September 2005. Patients with a cancer diagnosis prior to thyroid cancer were excluded. The patient's morbidity period was set from the date of initial diagnosis to the date of the other types of cancer, and for patients who did not develop other types of cancer, it was set until May 2021. For patients with DL and NAFLD simultaneously, the morbidity period was set from the date of diagnosis of the later disease. A nested case–control study was conducted to exclude lead-time bias in these patients. (Fig. 1A). To evaluate the risk of thyroid cancer on cancer occurrence in patients with metabolic disease and to identify factors that affect it, baseline characteristics such as age, sex, waist circumference, and body mass index (BMI) of the patients were assessed. Blood test results for glucose, aspartate aminotransferase (AST), alanine aminotransferase (ALT), total cholesterol, low-density lipoprotein (LDL) cholesterol, high-density lipoprotein (HDL) cholesterol, and triglyceride levels were collected. For each result, the value of the test period closest to the diagnosis period of NAFLD and DL was used. For patients with both diseases, values close to the time of diagnosis of the later diagnosed disease were used. The effect of thyroid cancer on the incidence of other types of cancer and the effects of the aforementioned clinical characteristics and blood test results were analyzed.

### Nationwide cohort

To generate the population-based cohort, we used data from the National Health Insurance Service-National Sample Cohort (NHIS-NSC), which has accumulated medical information for a population-based retrospective cohort based on a 2.2% representative sample of Korean citizens, covering all regions in Korea. The NHIS maintains the national records of all covered inpatient and outpatient visits, procedures, and medications. The sampling consisted of a systemic stratified random sample with proportional allocation within each stratum. We used person-level longitudinal NHIS-NSC registration and claims data available from 2002 to 2015, and patient groups were extracted using a method similar to that of our institutional data. To increase the accuracy of the patient group in the representative data, patients with medical records for which the corresponding diagnosis was entered twice or more were extracted. DL was extracted at least twice from confirmed patients with ICD 10 code E78.0–78.9. For NAFLD, patient groups were extracted using the inclusion and exclusion ICD 10 codes, as described in Supplementary Fig. 1^48^. Cancer occurrence in both groups was identified by extracting the patient group with thyroid cancer and matching age, sex, and index year 1:5. (Fig. 1B). To evaluate the risk of thyroid cancer on cancer occurrence in patients with metabolic diseases and to identify factors that affect it, the baseline characteristics and blood test results of the patients were collected. For each result, the value of the test period closest to the diagnosis period of NAFLD and DL was used. For patients with both diseases, values ​​close to the time of diagnosis of the later diagnosed disease were used.

This study was approved by the Institutional Review Board of Severance Medical Center (Seoul, South Korea) and conducted in accordance with the recommendations of the Institutional Review Board (4-2020-0777), which waived the requirement for informed consent because of the retrospective nature of this study.

### Statistical analysis

Data are presented as mean ± standard deviation for continuous variables and as proportions for categorical variables. Stratified Cox proportional hazard regression was performed to estimate the hazard ratio (HR) and corresponding 95% CIs. Statistical significance was set at *P* ≤ 0.05. All statistical analyses were performed using Statistical Product and Service Solutions, version 25.0 for Windows (SPSS Inc., Chicago, Illinois, USA) and SAS Enterprise Guide version 7.1 (SAS Institute Inc., Cary, NC).

## Supplementary Information

Supplementary Information.

## Data Availability

The datasets generated during and/or analysed during this study are available from the corresponding author upon reasonable request.
