# Supplementary material for: Impact of thyroid cancer on the cancer risk in patients with non-alcoholic fatty liver disease or dyslipidemia
Source: Sci Rep. 2023 Jan 19;13:1076. doi: 10.1038/s41598-023-28112-2 (PMC9852577; doi:10.1038/s41598-023-28112-2)
Supplement: Supplementary file 1 — Supplementary Information. [file 41598_2023_28112_MOESM1_ESM.pdf]

Impact of thyroid cancer on the cancer risk in patients with non-alcoholic fatty liver disease or dyslipidemia

Joon Ho, MD[1†], Eunhwa Kim, MS[2†], Myeongjee Lee, PhD[2], Inkyung Jung, PhD*[3], Young Suk Jo MD, PhD*[4], and Jandee Lee, MD, PhD*[1]

[1]Department of Surgery, Open NBI Convergence Technology Research Laboratory, Yonsei University College of Medicine, Seoul, South Korea;
[2]Biostatistics Collaboration Unit, Department of Biomedical Systems Informatics, Yonsei University College of Medicine, Seoul, South Korea;
[3]Division of Biostatistics, Department of Biomedical Systems Informatics, Yonsei University College of Medicine, Seoul, South Korea;
[4]Department of Internal Medicine, Open NBI Convergence Technology Research Laboratory, Yonsei University College of Medicine, Seoul, South Korea

[†] The first two authors equally contributed to this work.

***Correspondence and requests for materials should be addressed to:**

ijung@yuhs.ac (Jung I.); jandee@yuhs.ac (Lee J.); joys@yuhs.ac (Jo Y.S.) Tel: +82-2-2228-0752, Fax: +82-2-393-6884

This file includes

Supplementary Tables 1~5

Supplementary Figure 1

내과학교실/조*석/2023-01-10 07:00:34

Supplementary Table 1. Baseline characteristics of our institutional cohort

	**NAFLD or DL**	**NAFLD**	**DL**
No of patients	136,672	15,907	112,088
Age (years)	52.8 ± 12.1	49.6 ± 14.4	58.7 ± 12.0
Sex			
Men	68,830 (50.4%)	9,764 (61.4%)	54,155 (48.3%)
Women	67,842 (49.6%)	6,143 (38.6%)	57,933 (51.7%)
Thyroid cancer	1,289 (0.94 %)	147 (0.92 %)	1,058 (1.34 %)
F/U periods (months)	85.7 ± 59.5	84.5 ± 67.1	87.4 ± 58.7

NAFLD, non-alcoholic fatty liver disease; DL, dyslipidemia; F/U, follow-up

내과학교실/조*석/2023-01-10 07:00:34

Supplementary Table 2. Baseline characteristics of patients with NAFLD or dyslipidemia according to thyroid cancer in our institutional cohort after propensity score matching

	Thyroid cancer		P-value
	Absence (n=6,445)	Presence (n=1,289)	
Sex			
Male	1935 (30.02 %)	387 (30.02 %)	
Female	4510 (69.98 %)	902 (69.98 %)	
BMI	27.77 ± 6.55	29.28 ± 10.74	0.3154
Waist circumference	90.66 ± 18.65	90.08 ± 15.49	0.2348
Glucose	128.56 ± 70.42	138.35 ± 65.38	0.3163
Total cholesterol	173.82 ± 63.81	210.30 ± 98.17	< 0.0001
Triglyceride	154.21 ± 101.04	171.45 ± 90.99	0.9851
HDL	47.91 ± 14.25	45.65 ± 13.45	0.1080
LDL	101.43 ± 41.84	99.20 ± 40.27	0.4002
AST	37.43 ± 47.31	27.05 ± 14.42	< 0.0001
ALT	43.96 ± 70.68	29.55 ± 25.49	< 0.0001
γGT	65.82 ± 46.20	52.39 ± 44.75	< 0.0001

BMI: body mass index, HDL: high-density lipoprotein, LDL: low-density lipoprotein, AST: aspartate transaminase, ALT: alanine transaminase, γGT: gamma-glutamyl transpeptidase

내과학교실/조*석/2023-01-10 07:00:34

Supplementary Table 3. Cancer risk estimation by univariable stratified Cox regression analysis result after propensity score matching in our institutional cohort

	HR (95% CI)		
**Variables**	**NAFLD or DL**	**NAFLD**	**Dyslipidemia**
BMI	0.997 (0.992-1.003)	0.998 (0.987-1.009)	0.997 (0.991-1.004)
Waist	0.982 (0.955-1.010)	0.975 (0.942-1.010)	0.999 (0.951-1.050)
Glucose	1.004 (1.002-1.006)	1.004 (1.001-1.008)	1.003 (1.001-1.006)
Total cholesterol	0.997 (0.995-0.999)	0.996 (0.992-1.001)	0.996 (0.994-0.998)
Triglyceride	0.999 (0.998-1.000)	0.998 (0.996-1.000)	1.000 (0.999-1.001)
HDL	0.997 (0.989-1.005)	1.002 (0.988-1.017)	0.994 (0.985-1.004)
LDL	0.997 (0.994-0.999)	0.990 (0.993-1.002)	0.996 (0.993-1.000)
AST	0.998 (0.995-1.002)	1.000 (0.996-1.003)	0.999 (0.992-1.006)
ALT	0.997 (0.994-1.001)	0.999 (0.996-1.002)	0.999 (0.993-1.005)
γGT	1.001 (1.000-1.001)	1.002 (1.001-1.002)	1.001 (1.000-1.001)
Thyroid cancer (ref. = unexposed)	2.007 (1.597-2.522)	1.866 (1.072-3.249)	1.942 (1.491-2.529)

BMI: body mass index, HDL: high-density lipoprotein, LDL: low-density lipoprotein, AST: aspartate transaminase, ALT: alanine transaminase, γGT: gamma-glutamyl transpeptidase

내과학교실/조*석/2023-01-10 07:00:34

Supplementary Table 4. Cancer risk estimation by multivariable stratified Cox regression analysis result after propensity score matching in our institutional cohort

	HR (95% CI)		
	**NAFLD or DL**	**NAFLD**	**Dyslipidemia**
Glucose	1.003 (1.000-1.005)	1.003 (0.999-1.008)	1.003 (1.000-1.005)
Total cholesterol	0.997 (0.995-0.999)	0.996 (0.989-1.002)	0.997 (0.995-0.999)
LDL	0.999 (0.995-1.003)	0.998 (0.991-1.006)	1.000 (0.996-1.004)
γGT	1.001 (1.000-1.001)	1.002 (1.001-1.003)	1.001 (1.000-1.001)
Thyroid cancer (ref. = unexposed)	2.092 (1.546-2.829)	1.751 (1.038-3.658)	1.843 (1.296-2.620)

LDL: low-density lipoprotein, γGT: gamma-glutamyl transpeptidase

내과학교실/조*석/2023-01-10 07:00:34

Supplementary Table 5. Risk estimation of cancer in patients with NAFLD or dyslipidemia according to thyroid cancer in our institutional cohort after propensity score matching

	Thyroid cancer		
	Absence (n = 6,445)	Presence (n = 1,289)	
Cancer type	No. of cases (%)	No. of cases (%)	HR (95% CI)
**Overall**	**318 (4.93)**	**127 (9.85)**	**2.007 (1.597-2.522)**
Lip, tongue, mouth	1 (0.31)		
Oropharynx, nasopharynx	2 (0.63)	1 (0.78)	1.745 (0.152 – 20.068)
Esophagus	2 (0.63)	2 (1.57)	3.145 (0.431 – 22.937)
Stomach	31 (9.75)	9 (7.09)	0.826 (0.393 – 1.739)
Small intestine	1 (0.31)	1 (0.78)	2.610 (0.163 – 41.737)
Colon	30 (9.43)	7 (5.51)	0.597 (0.247 – 1.442)
Anus, anal canal		1 (0.78)	
Liver	42 (13.21)	8 (6.30)	0.523 (0.245 – 1.116)
Pancreas	8 (2.52)	6 (4.72)	2.010 (0.697 – 5.797)
Lung, bronchus	20 (6.29)	24 (18.90)	3.252 (1.795 – 5.892)
Thymus, mediastinum, heart	2 (0.63)	2 (1.57)	2.685 (0.378 – 19.080)
Bone, joints, soft tissue	11 (3.46)	15 (11.81)	3.697 (1.693 – 8.074)
Skin, melanoma	13 (4.09)	5 (3.94)	1.126 (0.399 – 3.178)
Breast	42 (13.21)	13 (10.24)	0.850 (0.456 – 1.585)
Uterus	17 (0.53)	1 (0.78)	0.166 (0.022 – 1.251)
Ovary	1 (0.31)	1 (0.78)	5.169 (0.323 – 82.743)
Prostate	8 (2.52)		
Kidney	22 (6.92)	7 (5.51)	0.827 (0.351 – 1.947)
Urinary bladder	14 (4.40)	1 (0.78)	0.196 (0.026 – 1.490)
Brain, CNS	9 (2.83)	3 (2.36)	0.926 (0.249 – 3.436)
Lymphoma	12 (3.77)	6 (4.72)	1.311 (0.492 – 3.494)
Multiple myeloma	6 (1.89)		
Leukemia	4 (1.26)	6 (4.72)	4.238 (1.183 – 15.176)
Others	20 (6.29)	8 (6.30)	1.090 (0.479 – 2.482)

Abbreviations: HR, hazard ratio; Total (%) = other primary cancer pts. / total pts. Cancer type (%) = specific type pts. / other primary cancer pts.

내과학교실/조*석/2023-01-10 07:00:34

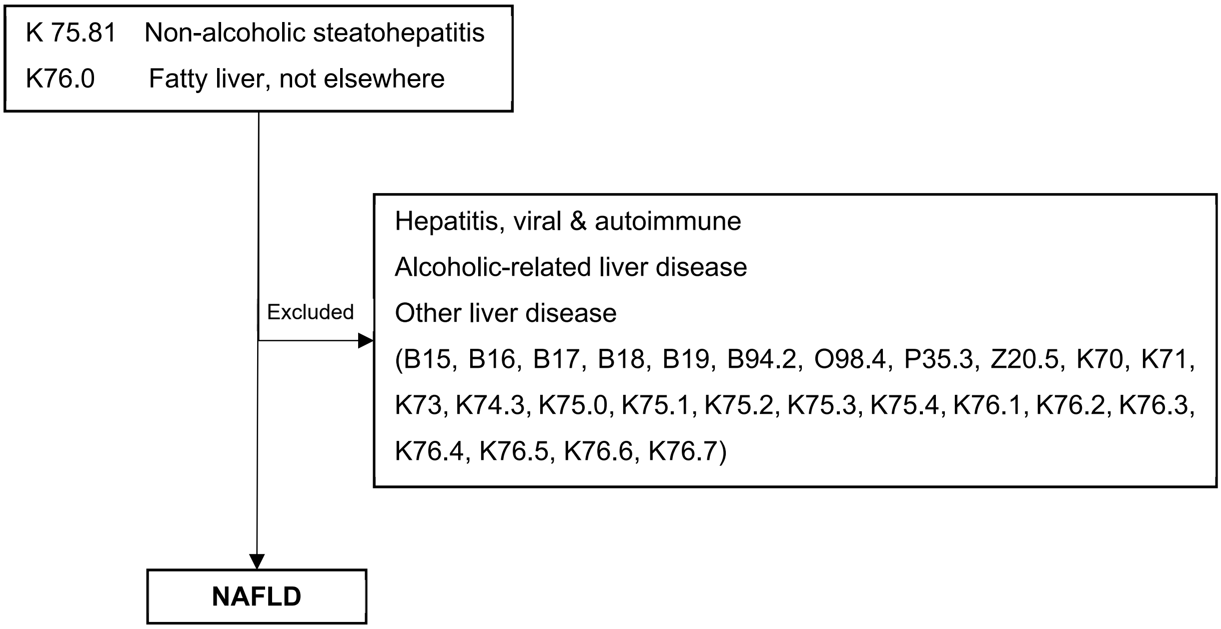

Supplementary Figure 1. Flowchart of identification of patients with NAFLD in the medical record system. NAFLD, non-alcoholic fatty liver disease.

내과학교실/조*석/2023-01-10 07:00:34
